# Motion Blurred Star Image Restoration Based on MEMS Gyroscope Aid and Blur Kernel Correction

**DOI:** 10.3390/s18082662

**Published:** 2018-08-13

**Authors:** Shiqiang Wang, Shijie Zhang, Mingfeng Ning, Botian Zhou

**Affiliations:** Research Center of Satellite Technology, Harbin Institute of Technology, Harbin 150080, China; 14B918056@hit.edu.cn (S.W.); 13B918064@hit.edu.cn (M.N.); zhoubotian@stu.hit.edu.cn (B.Z.)

**Keywords:** star image restoration, motion blur, MEMS gyroscope, blur kernel correction, interior point algorithm, scaled gradient projection

## Abstract

Under dynamic conditions, motion blur is introduced to star images obtained by a star sensor. Motion blur affects the accuracy of the star centroid extraction and the identification of stars, further reducing the performance of the star sensor. In this paper, a star image restoration algorithm is investigated to reduce the effect of motion blur on the star image. The algorithm includes a blur kernel calculation aided by a MEMS gyroscope, blur kernel correction based on the structure of the star strip, and a star image reconstruction method based on scaled gradient projection (SGP). Firstly, the motion trajectory of the star spot is deduced, aided by a MEMS gyroscope. Moreover, the initial blur kernel is calculated by using the motion trajectory. Then, the structure information star strip is extracted by Delaunay triangulation. Based on the structure information, a blur kernel correction method is presented by utilizing the preconditioned conjugate gradient interior point algorithm to reduce the influence of bias and installation deviation of the gyroscope on the blur kernel. Furthermore, a speed-up image reconstruction method based on SGP is presented for time-saving. Simulated experiment results demonstrate that both the blur kernel determination and star image reconstruction methods are effective. A real star image experiment shows that the accuracy of the star centroid extraction and the number of identified stars increase after restoration by the proposed algorithm.

## 1. Introduction

Being highly precise and highly reliable, a star sensor is one of the main devices of a celestial navigation system [1]. It is widely used in spacecraft attitude control [2], missile guidance [3], and ship and aircraft navigation [4,5], and is small in size and light in weight [6,7]. By executing star centroid extraction, star identification, and attitude estimation, the attitude information can be determined [8]. In spacecraft attitude determination, when the spacecraft is rapidly maneuvering, the relative motion between the star and imaging device during the exposure time makes the star spot move on the image plane, dispersing the energy of the stars, which causes motion blur. As a result, the signal to noise ratio of the star image decreases, and the accuracy of the centroid position of the star seriously reduces. Motion blur limits the performance of a star sensor under highly dynamic conditions, and can hardly be compensated effectively by traditional methods, such as mechanical, optical, or electronic approaches [9,10,11].

In the past years, many works have been done based on image processing, in order to improve the performance of star sensors under dynamic conditions. According to the working principle, they can be divided into three types.

Some researchers consider the location of the area with some special properties on the star strip as the position of the star spot. As in the literature [12], the position of a star is the centroid of the area between two negative correlation peaks on the gradient autocorrelation curve of the star strip. In the literature [13], the position of the corner points on the strip take the place of the position of the star strip. These two methods can only be performed well in the case of strips with uniform energy distribution. From the perspective of energy accumulation, the energy of a strip is accumulated from one endpoint to another along a specific direction [14,15,16]. However, in this type of method, the direction and length of the accumulation should be known precisely.

There is an offset between the centroid of the strip and its actual position. In this case, some researchers draw support from other sensors to obtain this offset. In the literature [17], the author derives the offset between the centroid of the strip and the actual position of the strip according the relationship between the angular velocity and the motion of the star spot in the image. A deeply-coupled method with a gyroscope and star sensor is proposed by the authors of [18], in which the error of the gyroscope and the offset of the star spot position are estimated simultaneously by an extended Kalman filter. This type of method is easy to implement, but the performance is limited by the cooperative sensors.

The most common method is image restoration. There are two problems that should be solved in star image restoration. One is blur kernel determination, and the other is the image reconstruction method. A large number of work has been done regarding these two issues. For blur kernel determination, it is assumed that the star strips are composed of a set of parallel lines, so the Radon transform can be used to obtain the length and angle of the star strip, and then the blur kernel is calculated [19,20]. Once the star strip cannot be considered as a set of parallel lines (this case often happens in boresight rotation), error will be introduced to the blur kernel. In the literature [21,22,23,24], the motion model of the star sensor is used to determine the blur kernel. These methods are very effective, and they can determine the blur kernel in any motion. However, the methods cannot work well when the inputs of the motion model are inaccurate. For star image reconstruction, there are three types of common methods. The first one is the Wiener filter [25,26,27]. Quan and Zhang [25] and Zhang et al. [26] use weight allocation between the background pixels and strip pixels to reduce the ring phenomenon in the restored image. Wang et al. [27] use the idea of descending dimension to decompose the two-dimensional Wiener filter into two one-dimensional (1D) Wiener filters according to the movement of the pixel on the image plane. This method can double the speed of calculation. Even though the Wiener filter can run very fast, it will amplify the image noise. The Richardson–Lucy (RL) method can effectively suppress noise [19,20,21,22]. For the shortcoming of the long, heavy computation for the RL method, Jiang et al. [19] adopt vector extrapolation to accelerate the RL method. However, the ringing phenomenon is enhanced with iteration by using the RL method. To overcome the ringing phenomenon, Zhang et al. [24] use a regularization method. A mathematical model of motion blur with a regularization item is deduced according to the sparsity of the star image, then a convex optimization is performed to restore the blurred image. This method is sensitive to the errors of the blur kernel.

As discussed above, the Radon transform can only be used on the condition that there is no rotation along the boresight. As a star strip is mainly caused by the rotation of the star sensor, the MEMS gyroscope can be used at any angular velocity (including boresight rotation) for angular velocity measurement, because it is small in size, light in weight, and easy to integrate. In this study, a MEMS gyroscope is used as an assistant instrument for blur kernel determination. In order to mitigate the influence of the bias of the MEMS gyroscope and the installation deviation between the gyroscope and the star sensor on the blur kernel, the structure information of the star strip is extraction using Delaunay triangulation. By utilizing the structure information, the preconditioned conjugate gradient interior point method is used to correct the blur kernel from the gyroscope. For star image reconstruction, the current method used for star image reconstruction is either time-consuming or results in unwanted artifacts. The maximum likelihood estimate with scaled gradient projection (SGP) is used to reconstruct the star image to improve the reconstruction speed and quality.

This paper is organized as follows: The introduction is given in Section 1. The problem introduced by motion blur and the mathematical model of star image restoration are outlined in Section 2. The overall scheme of the proposed algorithm is presented in Section 3. The blur kernel determination method is described in detail in Section 4. Section 5 reports the star image reconstruction. In Section 6, the results of simulation and real image experience are shown. At the end, conclusions are drawn in Section 7.

## 2. Problem Formulation

### 2.1. Problem Introduced by Motion Blur

**Figure 1:**
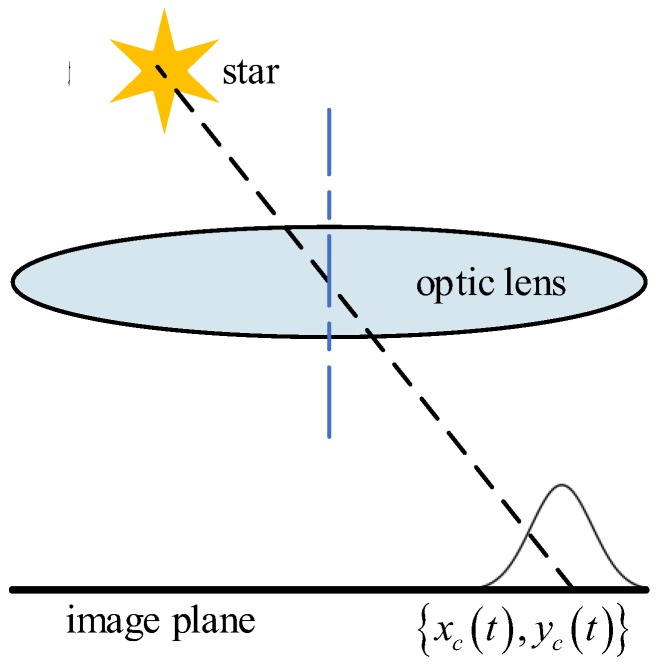
Imaging process of a star spot under static conditions.

**Figure 2:**
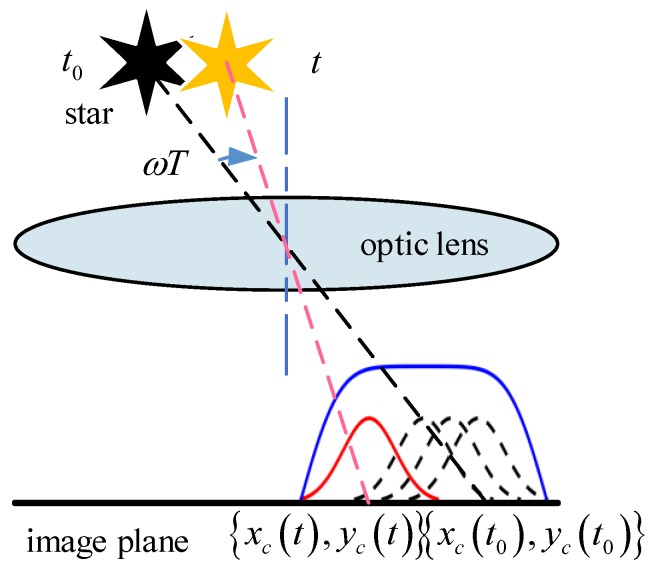
Imaging process of a star spot under dynamic conditions.

Extracting the centroid of a star spot in a star image accurately is the necessary precondition for attitude, which is determined by the star sensor. Like camera imaging, the star spot in a star image is generated by accumulating star energy in a certain exposure time. Under static conditions the energy of a star spot can be described as a two-dimension Gaussian distribution [28], as shown in Figure 1. When the star sensor is under dynamic conditions, star spots move on the image plane, and a star stripe is generated, as shown in Figure 2. This process leads to the motion blur of the star image. In this case, the energy of the star spot disperses over the star strip and makes the strip dim [29]. When the rotation of the star sensor becomes faster and faster, more and more stars in the image will break into pieces or submerge into the background. This causes the centroid of the star to be shifted by several pixels and reduces the number of detectable stars, which makes it difficult for star identification and attitude determination. Therefore, it is necessary to restore the blurred star image and reconstruct the star spot from the star strip.

### 2.2. Mathematical Model of Star Image Restoration

According to the authors of [30], motion blur of a star image is one type of image degradation. It is assumed that the degeneration process is linear space invariant, and that the process of image degeneration can be modeled by the convolution operation between the original image and the degradation function, as follows:

(1) Y=X⊗K+η

where $Y\in {\mathbb{R}}^{n\times n}$ is the blur star image that is obtained by the star sensor, $X\in {\mathbb{R}}^{n\times n}$ is the original image, $K\in {\mathbb{R}}^{i\times i}$ is the blur kernel that is the matrix form of the degradation function, and ⊗ is the convolution operation. In this paper, it describes the motion of the star spot in the image plane during the exposure time. $\eta \in {\mathbb{R}}^{m\times n}$ is the image noise.

On the contrary, image restoration uses prior information of image degradation to restore the blur image Y, and the result of restoration $\hat{X}$ is the estimation of the original image. In this paper, the prior information of the image degradation is K, so the star image restoration is a non-blind deconvolution. By representing a two-dimensional image $X\in {\mathbb{R}}^{n\times n}$ as a vector $x={\left[x_{1},x_{2},...,x_{N}\right]}^{T}\in {\mathbb{R}}^{N},N=n^{2}$, the entirety of ***X*** is stacked column by column. $A\in {\mathbb{R}}^{N\times N}$ is the circulate matrix of the blur kernel ***K***. $b={\left[b_{1},b_{2},...,b_{N}\right]}^{T}\in {\mathbb{R}}^{N},N=n^{2}$ is the same operation of ***Y*** as ***X***.

Therefore, the image restoration can be modeled as a minimum problem, as follows:

(2) $$\begin{array}{l} \hat{x}=\arg\underset{x}{\min}\frac{1}{2}{\left\|Ax-b\right\|}_{2}^{2}\\ \mathrm{subject}\;\mathrm{to}\begin{array}{cc} & x\geq 0 \end{array} \end{array}$$

where ${\left\|\cdot \right\|}_{2}$ is the ℓ2-norm. Equation (2) is the mathematical model of the star image restoration, ***A*** (or K) plays a key role in restoration; its accuracy will directly affect the result of the restoration.

### 2.3. Mathematical Model of the Blur Kernel

The energy distribution model of a star strip can describe the blur process explicitly, so the blur kernel can be determined by this model.

According to the authors of [28], the star spot on the image plane is static under static conditions. It is assumed that the energy of a star spot can be described as a two-dimension Gaussian distribution, so the function of its energy distribution is as follows:

(3) $$f\left(x,y\right)=\frac{\Phi T}{2\pi \rho^{2}}\exp\left[-\frac{{\left(x-x_{c}\right)}^{2}+{\left(y-y_{c}\right)}^{2}}{2\rho^{2}}\right]$$

where Φ is the incident flux of the star in the image plane, *T* is the exposure time, ρ is the variance of the Gaussian distribution, and $\left(x_{c},y_{c}\right)$ represents the coordinates of the center of the star spot.

The position of a star spot on the image plane will change with time in a dynamic condition, and the function of its energy distribution is as follows:

(4) $$g\left(x,y\right)=\int_{0}^{T}\frac{\Phi }{2\pi \rho^{2}}\exp\left\{-\frac{{\left[x-x_{c}\left(t\right)\right]}^{2}+{\left[y-y_{c}\left(t\right)\right]}^{2}}{2\rho^{2}}\right\}dt$$

The integral in Equation (4) expresses that the process of the star spot as it moves on the image plane according to the motion trajectory $\left\{x_{c}\left(t\right),y_{c}\left(t\right)\right\}$, which generates a strip. Therefore, the motion trajectory in Equation (4) is the mathematical model of the blur kernel.

## 3. Overview of the Proposed Method

**Figure 3:**
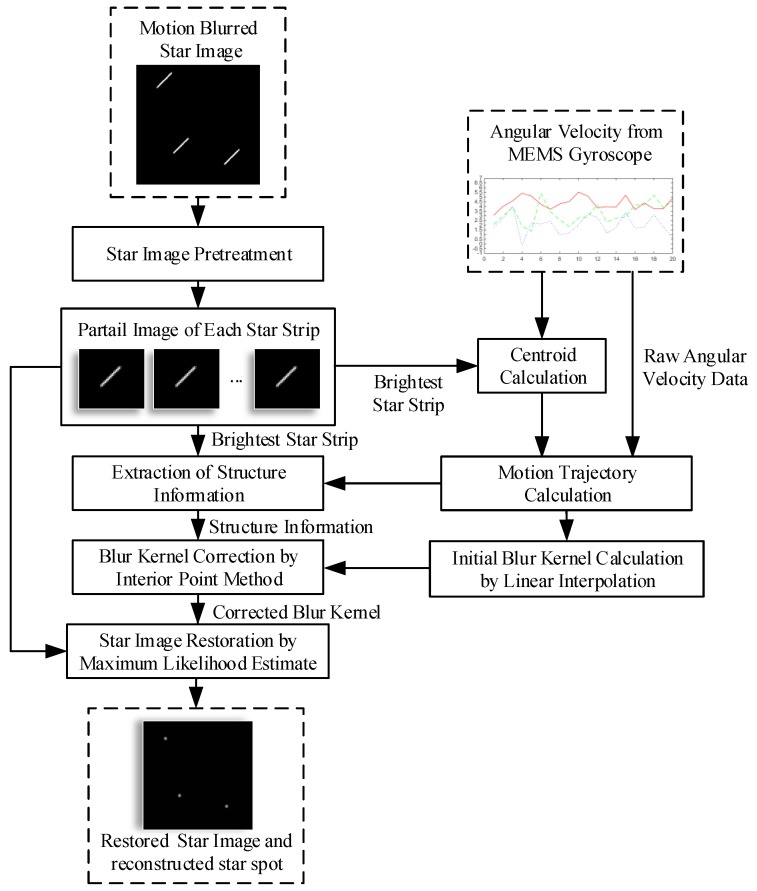
The flowchart of proposed method

As mentioned above, a blur kernel is necessary to restore the blurred star image. The blur kernel is concerned with motion trajectory. As a star strip is mainly caused by the rotation of the star sensor, a MEMS gyroscope can be used as an assistant device to obtain the blur kernel at any angular velocity. In ideal conditions, the blur kernel can be deduced from the precious angular velocity. As the bias and installation deviation of the gyroscope can affect the accuracy of the blur kernel, a correction process is need. The strip in the star image is the intuitionistic behavior of motion blur, the structure information of the star strip is used to correct the blur kernel from the gyroscope in this paper. Afterwards, a fast star spot reconstruction is carried out with the corrected blur kernel. The flow chart is shown in Figure 3.

Firstly, it is assumed that the brightest star strip can be detected. Before the proposed method is performed, denoising and star segmentation operations are required to obtain partial images, and each partial image contains a star strip. The motion trajectory points can be calculated with the centroid of the brightest strip and the angular velocity data from the gyroscope. With these trajectory points, the blur kernel is generated by linear interpolation. The result of this step is the initial blur kernel.

Then, as the initial blur kernel is not precise, a correction process is needed. The strip in the star image is the intuitionistic behavior of motion blur, and the structure information of the star strip can be used to correct the blur kernel. In this paper, the structure information of the brightest star strip is extracted by Delaunay triangulation. The brightest strip may break into pieces when the rotation is fast enough. The pieces that belong to the same star strip are determined aided by the motion trajectory. After the structure information is extracted, a preconditioned conjugate gradient interior point method is used to obtain an optimized blur kernel with this structure information. This process achieves the purpose of correction. The result of the correction is the corrected blur kernel.

Finally, a maximum likelihood estimate is used to reconstruct the star image with the corrected blur kernel. To improve the reconstruction speed and reconstruction quality without introducing significant additional costs and unwanted artifacts, SGP is used in the reconstruction process. This completes the star image restoration process.

## 4. Blur Kernel Determination

### 4.1. Initial Blur Kernel Calculation Aided by the Gyroscope

During exposure, the trajectory of the star spot on image plane reflects the process of motion blur, so, the trajectory contains information about the blur kernel. 

For the sake of convenience, the exposure time is divided into several short time intervals, Δt. Let $A^{t}$ be the attitude matrix of star sensor at present moment, the observed vector of a star in star sensor coordinates is defined as $w_{i}$, and $v_{i}$, is the corresponding vector in inertial coordinate, so the relationship of these variance is as follows:

(5) $$\left\{ \begin{array}{c} w_{i}^{t}=A^{t}v_{i}^{t}\\ w_{i}^{t+\Delta t}=A_{t}^{t+\Delta t}A_{t}^{t}v_{i}^{t} \end{array}\right.$$

where $A_{t}^{t+\Delta t}$ is the attitude matrix of the next moment, it can be calculated from Equation (5), as follows:

(6) $$A_{t}^{t+\Delta t}=I-{\tilde{\omega }}^{t}\Delta t=I-\left[\begin{array}{ccc} 0 & -\omega_{z}^{t} & \omega_{y}^{t}\\ \omega_{z}^{t} & 0 & -\omega_{x}^{t}\\ -\omega_{y}^{t} & -\omega_{x}^{t} & 0 \end{array}\right]\Delta t$$

where $\tilde{\omega }$ is the cross-product matrix populated with the components of the angular velocity vector ω. Then, the relationship between $w_{i}^{t}$ and $w_{i}^{t+\Delta t}$ in Equation (7) can be obtained in terms of Equations (5) and (6) as follows:

(7) $$w_{i}^{t+\Delta t}=A_{t}^{t+\Delta t}w_{i}^{t}$$

where $w_{i}^{t}$ can be calculated with the star position in the image plane coordinates. Suppose that during the period of Δt, the angular velocity is constant. For the *i*th star in the image plane, its trajectory can be calculated by the following:

(8) $$\left\{ \begin{array}{l} x_{i}^{t+\Delta t}=\frac{x_{i}^{t}+y_{i}^{t}\omega_{z}^{t}\Delta t+f\omega_{y}^{t}\Delta t}{\left(-x_{i}^{t}\omega_{y}^{t}\Delta t+y_{i}^{t}\omega_{x}^{t}\Delta t\right)/f+1}\\ y_{i}^{t+\Delta t}=\frac{y_{i}^{t}-x_{i}^{t}\omega_{z}^{t}\Delta t-f\omega_{x}^{t}\Delta t}{\left(-x_{i}^{t}\omega_{y}^{t}\Delta t+y_{i}^{t}\omega_{x}^{t}\Delta t\right)/f+1} \end{array}\right.$$

In Equation (8), the focus length *f* is much greater than $-x_{i}^{t}\omega_{y}^{t}\Delta t+y_{i}^{t}\omega_{x}^{t}\Delta t$, so the denominator of Equation (8) is approximate to 1, and Equation (8) can be rewritten as follows:

(9) $$\left\{ \begin{array}{l} x_{i}^{t+\Delta t}=x_{i}^{t}+y_{i}^{t}\omega_{z}^{t}\Delta t+f\omega_{y}^{t}\Delta t\\ y_{i}^{t+\Delta t}=y_{i}^{t}-x_{i}^{t}\omega_{z}^{t}\Delta t-f\omega_{x}^{t}\Delta t \end{array}\right.$$

From Equation (9), the motion trajectory of a star spot on the image plane can be obtained during the rotation. Thus, to calculate the position of the star spot in the image plane before exposure, a set of angular velocities and time intervals during exposure are variables that must necessarily be known. As the position of the star spot is difficult to obtain when the exposure begins, herein, the centroid of the star strip is chosen as a substitute. Suppose this centroid can be taken as the position when time $t_{m}$, which is exactly at half of the exposure time. Then, the trajectory after $t_{m}$ can be calculated by Equation (9), and the trajectory before $t_{m}$ can be calculated by Equation (10), as follows:

(10) $$\left\{ \begin{array}{l} x_{i}^{t+\Delta t}=x_{i}^{t}-y_{i}^{t}\omega_{z}^{t}\Delta t-f\omega_{y}^{t}\Delta t\\ y_{i}^{t+\Delta t}=y_{i}^{t}+x_{i}^{t}\omega_{z}^{t}\Delta t+f\omega_{x}^{t}\Delta t \end{array}\right.$$

**Figure 4:**
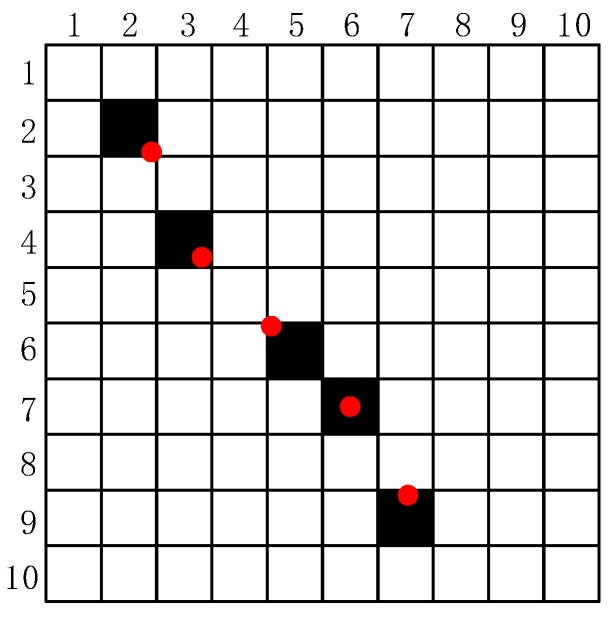
The trajectory points in image.

Suppose there are *n* trajectory points $P=\left\{\left(x_{i}^{t+\Delta t},y_{i}^{t+\Delta t}\right),\left(x_{i}^{t+2\Delta t},y_{i}^{t+2\Delta t}\right),...,\left(x_{i}^{t+n\Delta t},y_{i}^{t+n\Delta t}\right)\right\}$ after exposure. The coordinates of each trajectory point are not integers, so a rounding operation is done and expresses them in a matrix. As shown in Figure 4, the red dots are the actual position of the trajectory points, the black squares are their position by rounding operation, and the white squares are zeros elements. As the trajectory points in the matrix are disconnected, and the value of non-zero elements in the matrix are unknown, a post-process is needed.

To solve the problem above, the adjacent two trajectory points of ***P*** are divided into several interval points. The coordinates of each interval point are calculated by using the linear interpolation method. After a round operation, if there are $M_{k}$ interval points at the position of a certain pixel, the value of this pixel is $M_{k}$. The details of are shown in **Algorithm 1**.
**Algorithm 1:** linear interpolation between adjacent two trajectory points
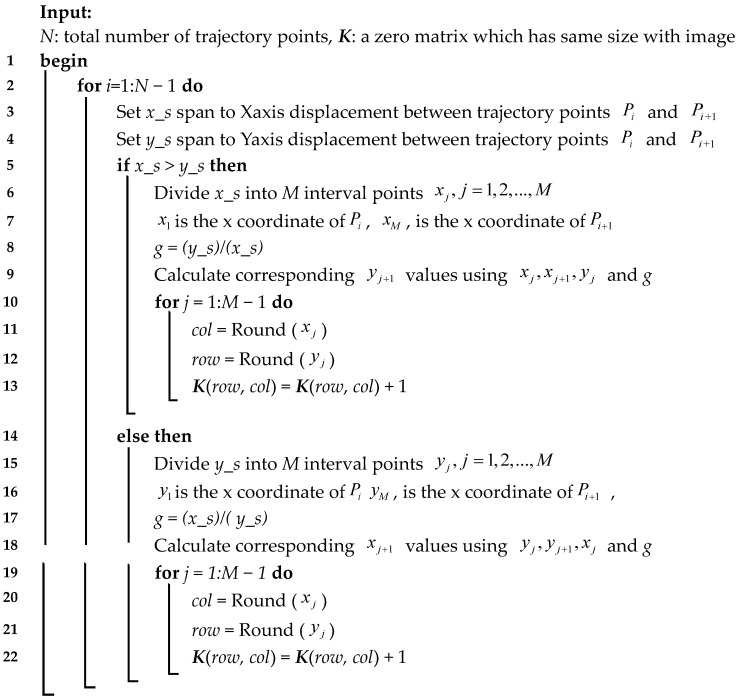


After the zero elements around the trajectory points in ***K *** are deleted, the initial blur kernel is obtained.

### 4.2. Structure Information Extraction of the Star Strip

The bias of the gyroscope data and the installation deviation between the gyroscope and the star sensor can affect the accuracy of the blur kernel from the gyroscope. The star strip is the intuitionistic behavior of motion blur, so, the structure information of the strip is used to correct the blur kernel. As the strip is an elongated shape, its centerline contains the structure information. The skeleton is an important feature in describing the geometrical shapes of the star strips. It is a one-pixel width polyline that crosses the center of the region. Thus, the skeleton of the strip can be considered as the centerline and the structure information of the star strip can be obtained by skeletonization.

As the structure information is extracted in the binary image, it is assumed that the binarization of the partial star image has been done. If the strip breaks into pieces, a pretreatment is need before skeletonization. The pieces that are crossed by the same motion trajectory belong to the same strip. Then, morphological dilation and erosion are used to connect these pieces to a whole strip.

**Figure 5:**
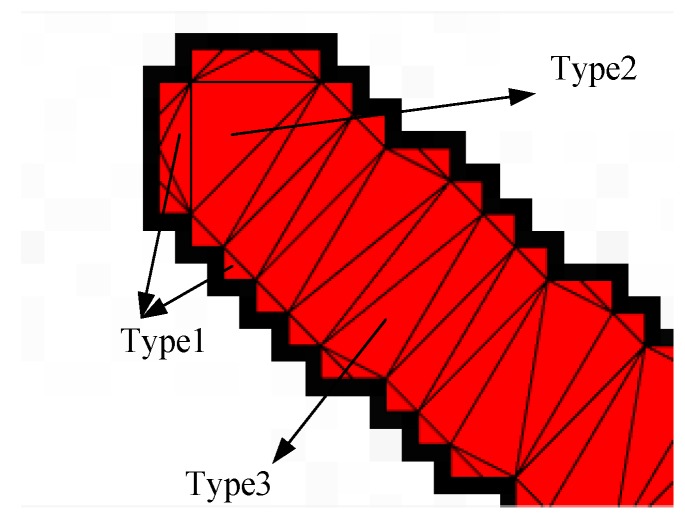
Triangle mesh of a star strip.

To obtain the skeleton of a star strip quickly, a meshing method is adopted. As triangles have a great deal of flexibility in the representation of geometric shapes, they are chosen as the element of the mesh. Delaunay triangulation is the most well-developed and effective method in all two-dimensional planar triangulation methods. It has the advantage of simplicity for implementation, little memory expense, and it can adapt to a variety of irregular shapes. Therefore, it is suitable for the skeletonization of a star strip. The detail of the Delaunay triangulation can be found in the literature [31]. For the triangle mesh of star strip, there are three types of triangle after triangulation, as shown in Figure 5.

The first type is small-sized triangles, which mainly appear at the border of the strip area. The second type is the end triangles, which are large in size, and mainly appear at both ends of the strip area. The third type is structure triangles, which form the body of the strip. The first type of triangles has no contribution to the skeletonization, but do the opposite. So, they should be ignored.

**Figure 6:**
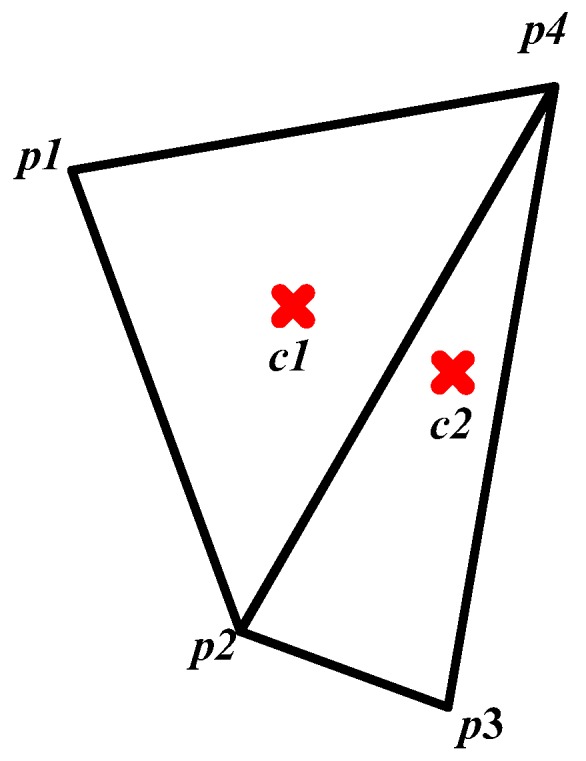
Skeleton points of a triangle mesh.

The skeleton is formed by the local symmetry points of the local area [32]. Thus, to each triangle, a point that reflects the local symmetry should be selected as the skeleton. As shown in Figure 6, the end triangle is similar to the equilateral triangle, so its circumcenter, *c*1, is the skeleton point, as follows:

(11) $$c1=\frac{\left(p1+p2+p4\right)}{3}$$

The structure triangle is similar to the isosceles triangle; its height is much longer than its base-side, so the skeleton point *c*2 is the middle of the line which connects the midpoints of two hypotenuses, as follows:

(12) $$c2=\frac{1}{2}\left(\frac{\left(p2+p4\right)}{2}+\frac{\left(p3+p4\right)}{2}\right)$$

Expressing the skeleton points in a matrix ***T***, the value of each matrix element is determined as follows:

(13) $$T\left(i,j\right)=\left\{ \begin{array}{ll} I(i,j), & \mathrm{if}\;point(j,i)\;\mathrm{is}\;\mathrm{skeleton}\;\mathrm{point}\\ 0, & \mathrm{else} \end{array}\right.$$

where ***I*** is a partial star image that contains only one star strip. In the next section, a correction method is adopted to correct the blur kernel with matrix ***T***.

### 4.3. Blur Kernel Correction with Structure Information of a Star Strip

The blur kernel correction can be expressed as a small-scaled convex optimization model, as follows:

(14) $$\mathrm{minimize}\;{\left\|Ax-y\right\|}_{2}^{2}+\varphi {\left\|x\right\|}_{1}$$

where $x\in R^{n}$ is the vector form of the blur kernel to be estimated, ***A*** is the data matrix, $y\in R^{m}$ is the observed item, here, the vector form of matrix ***T***. φ≥0 is the regularization coefficient.

Equation (14) is a ℓ1-regularized least square problem, and the objective function is convex, but not differentiable. There are no analytic formulae or expressions for the optimal solution; its solution must be computed numerically.

To solve Equation (14), the interior point method is adopted. Firstly, a logarithmic barrier for the bound constraints $x_{i}\geq 0$ is defined, replacing the inequality constraint, as follows:

(15) $$\begin{array}{c} \Phi \left(x\right)=-\frac{1}{t}\sum_{i=1}^{n}\log x_{i}\\ dom\Phi =\left\{x\in {\mathbb{R}}^{n}|x_{i}>0,i-1,2,...,n\right\} \end{array}$$

Substituting Equation (15) into Equation (14), when x≥0, the 1-norm item ${\left\|x\right\|}_{1}$ in Equation (14) can be replaced by $\sum_{i=1}^{n}x_{i}$ Therefore, the objective function can be transformed into the following:

(16) $$\phi_{t}\left(x\right)=t{\left\|Ax-y\right\|}_{2}^{2}+t\varphi \sum_{i=1}^{n}x_{i}-\sum_{i=1}^{n}\log x_{i}$$

where *t* > 0 is the penalty factor. Equation (16) can be minimized by the Newton method. For any *t* > 0, the central path consists of the unique minimizer, $x^{★}(t)$, of the convex function of Equation (16). 

For faster convergence, a preconditioned conjugate gradient algorithm is used to compute the search step as the exact solution to the Newton system, which avoids the calculation of the Hessian, as follows:

(17) $$\Delta x=-{\left(2tA^{T}A+D\right)}^{-1}g$$

where, $D=diag\left(1/x_{1}^{2},\cdots ,1/x_{n}^{2}\right),g=2tA\left(Ax-y\right)+\left[\begin{array}{c} t\varphi_{1}-1/x_{1}\\ \vdots \\ t\varphi_{n}-1/x_{n} \end{array}\right]$.

As a stopping criterion, a duality gap is defined as the lower bound of the difference between the primal function value and the dual function value, as Equation (18), as follows:

(18) $$\eta ={\left\|Ax-y\right\|}_{2}^{2}+\varphi {\left\|x\right\|}_{1}-G\left(\upsilon \right)$$

where G(υ) is the dual function, and υ=2s(Ax-y) is the dual feasible point, which can be expressed, respectively, as follows:

(19) $$G\left(\upsilon \right)=-\frac{1}{4}\upsilon^{T}\upsilon -\upsilon^{T}y$$

(20) υ=2s(Ax-y)

The detail of the dual function is in Appendix A. In Equation (20), *s* is the step length, and can be determined by the following:

(21) $$s=\min\left\{\varphi /\left|2{\left(A^{T}Ax\right)}_{i}-2y_{i}\right|,i=1,2,...,m\right\}$$

The lower bound of the optimal value of the primal function can be given through the dual feasible point. Thus, the optimal value of G(ν) is the optimal lower bound of the primal function. According to weak duality, the ratio of duality gap to the dual function value is the optimal upper bound, as follows:

(20) $$\frac{f(x)-p^{★}}{p^{★}}\leq \frac{\eta }{G\left(\nu \right)}$$

where $p^{★}$ is the optimal value of the objective function, and *f*(***x***) is the primal objective computed with the point. When $\frac{\eta }{G\left(\nu \right)}<\varepsilon$, the iteration is stopped, or *t* is updated by the following rule, and continues iterating as follows:

(23) $$\{\begin{array}{cc} \begin{array}{l} t=\max\left\{\mu \min\left\{2n/\eta ,t\right\},t\right\},\\ t=t, \end{array} & \begin{array}{l} s\geq s_{\min}\\ s<s_{\min} \end{array} \end{array}$$

where μ>1 is the scale factor and $s_{\min}\in \left(0,1\right]$.

The whole process to solve Equation (14) is shown in **Algorithm 2**.
**Algorithm 2:** preconditioned conjugate gradient interior point algorithm
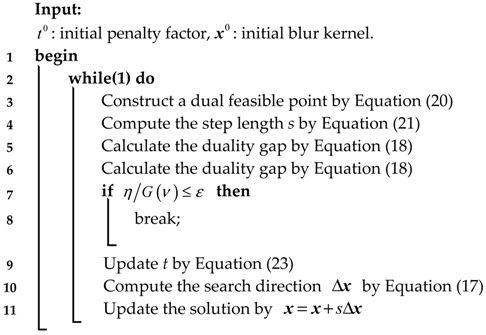


## 5. Star Image Reconstruction

In Equation (2), its objective function $J\left(x\right)=\frac{1}{2}{\left\|Ax-b\right\|}_{2}^{2}$ is a continuously differentiable convex function, and the constraints force the non-negativity of the solution. It can be solved by a maximum likelihood estimate. ***b*** is the sum of two terms, the blur image $b_{g}$ and Poisson noise $b_{\eta }$. Thus, the maximum likelihood estimate to solve the star image reconstruction can be transformed to a minimized problem of the Kullback–Leibler divergence [33,34], the objective function is as follows:

(24) $$f\left(x\right)=\sum_{i=1}^{N}\left(b_{i}\ln\frac{b_{i}}{{\left(Ax\right)}_{i}}+{\left(Ax\right)}_{i}-b_{i}\right)$$

To improve the calculation speed without introducing an additional cost, here, a SGP is used to solve the minimized problem of Equation (24). The gradient and hessian of f(x) are as follows:

(25) $$\nabla f\left(x\right)=A^{T}e-A^{T}U^{-1}b$$

(26) $$\nabla^{2}f\left(x\right)=A^{T}YU^{-2}A$$

In Equations (25) and (26), $e\in R^{N}$ is a vector whose elements are all equal to one, and Y=diag(b) and U=diag(Ax) are diagonal matrices. In the *k*th iteration, the solution is updated by the following:

(27) $$x^{\left(k+1\right)}=x^{\left(k\right)}+\lambda_{k}d^{\left(k\right)}$$

(28) $$d^{\left(k\right)}=y^{\left(k\right)}-x^{\left(k\right)}$$

where $\lambda_{k}\in \left(0,1\right]$ is the line search parameter, used to reduce the size of the step along the feasible descent direction $d^{\left(k\right)}$. It can be determined by a non-monotone strategy [35]. $y^{\left(k\right)}$ is with respect to the elements of the matrix, ***D***, in a non-negative quadrant, when ***x*** ≥ 0, can be written as follows:

(29) $$y^{\left(k\right)}=\{\begin{array}{cc} \begin{array}{l} x^{\left(k\right)}-\alpha_{k}D_{k}\nabla f\left(x^{\left(k\right)}\right),\\ 0, \end{array} & \begin{array}{l} \mathrm{if}\;x^{\left(k\right)}-\alpha_{k}D_{k}\nabla f\left(x^{\left(k\right)}\right)\geq 0\\ \mathrm{else} \end{array} \end{array}$$

In Equation (29), $\alpha_{k}$ is the step-length, $0<\alpha_{\min}<\alpha_{k}<\alpha_{\max},\forall k$ $D_{k}=diag\left(d_{1}^{\left(k\right)},d_{2}^{\left(k\right)},...,d_{N}^{\left(k\right)}\right)$ is a diagonal scaling matrix, with its elements satisfying the following:

(30) $$\frac{1}{L}\leq d_{i}^{\left(k\right)}\leq L,\;i=1,2,...,N\;\forall k,L\geq 1$$

The update rule of scaling matrix is as follows:

(31) $$\begin{array}{l} d_{i}^{\left(k\right)}=diag\left(\min\left\{L,\max\left\{\frac{1}{L},x_{i}^{\left(k\right)}\right\}\right\}\right),i=1,2,...,N\\ L=\sqrt{1+10^{10}/k^{2}} \end{array}$$

For the step-length parameter, a selection strategy derived by an updating rule is introduced in the literature [36]. This step-length selection is based on an adaptive alternation of the two Barzilai and Borwein (BB) rules [37], which, in the case of the scaled gradient directions, are defined by the following:

(32) $$\alpha_{k}^{\mathrm{BB}1}=\frac{{\left(s^{\left(k-1\right)}\right)}^{T}D_{k}^{-1}D_{k}^{-1}s^{\left(k-1\right)}}{{\left(s^{\left(k-1\right)}\right)}^{T}D_{k}^{-1}w^{\left(k-1\right)}},\alpha_{k}^{\mathrm{BB}2}=\frac{{\left(s^{\left(k-1\right)}\right)}^{T}D_{k}^{}w^{\left(k-1\right)}}{{\left(w^{\left(k-1\right)}\right)}^{T}D_{k}^{}D_{k}^{}w^{\left(k-1\right)}}$$

In Equation (32) $s^{\left(k-1\right)}=x^{\left(k\right)}-x^{\left(k-1\right)}$, $w^{\left(k-1\right)}=\nabla f\left(x^{\left(k\right)}\right)-\nabla f\left(x^{\left(k-1\right)}\right)$. The step-length is determined by the following:

(33) $$\alpha_{k}=\{\begin{array}{cc} \begin{array}{l} \min\left\{\alpha_{j}^{\left(2\right)},j=\max\left\{1,k-M_{\alpha }\right\},...,k\right\};\tau_{k+1}=\tau_{k}*0.9,\\ \alpha_{k}^{\left(1\right)};\tau_{k+1}=\tau_{k}*1.1, \end{array} & \begin{array}{l} \alpha_{k}^{\left(2\right)}/\alpha_{k}^{\left(1\right)}\leq \tau_{k}\\ \alpha_{k}^{\left(2\right)}/\alpha_{k}^{\left(1\right)}>\tau_{k} \end{array} \end{array}$$

where τ∈(0,1) is the alternating parameter of $\alpha_{k}^{}$, $M_{\alpha }$ is a positive integer, which controls the memory length of $\alpha_{j}^{\left(2\right)}$, and $\alpha_{k}^{\left(1\right)}$ and $\alpha_{k}^{\left(2\right)}$ are calculated by the following:

(34) $$\alpha_{k}^{\left(1\right)}=\{\begin{array}{cc} \begin{array}{l} \max\left\{\alpha_{\min},\min\left\{\alpha_{k}^{\mathrm{BB}1},\alpha_{\max}\right\}\right\},\\ \alpha_{\max}, \end{array} & \begin{array}{l} \mathrm{if}\;{\left(s^{\left(k-1\right)}\right)}^{T}D_{k}^{-1}w^{\left(k-1\right)}\geq 0\\ \mathrm{else} \end{array} \end{array}$$

(35) $$\alpha_{k}^{\left(2\right)}=\{\begin{array}{cc} \begin{array}{l} \max\left\{\alpha_{\min},\min\left\{\alpha_{k}^{\mathrm{BB}2},\alpha_{\max}\right\}\right\},\\ \alpha_{\max}, \end{array} & \begin{array}{l} \mathrm{if}\;{\left(s^{\left(k-1\right)}\right)}^{T}D_{k}^{}w^{\left(k-1\right)}\geq 0\\ \mathrm{else} \end{array} \end{array}$$

The whole process to solve the minimization of Equation (24) is shown in **Algorithm 3**.
**Algorithm 3:** Image reconstruction by SGP
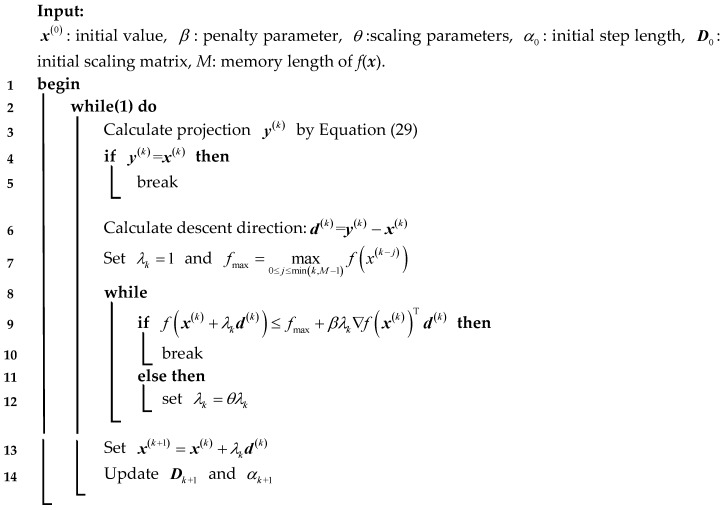


## 6. Experiment and Results

The experiments in this section aim to show the performance of the proposed method. All of the experiments are conducted with MATLAB on a computer with Intel(R) Core(TM) i5-480M processor and 8 GB of random-access memory (RAM).

### 6.1. Simulated Experiment

There are three simulated experiments. Firstly, the comparison of different blur kernel determination methods is conducted to verify the performance of the proposed blur kernel determination method. Then, the star images are reconstructed based on the blur kernels which are determined by different methods to further verify the superiority of the proposed blur kernel determination method. All of the images are reconstructed by the same reconstruction method. Finally, the comparison of the different reconstruction methods with the same blur kernel is conducted to verify the performance of the proposed star image reconstruction method.

#### 6.1.1. Simulation Condition

In this experiment, the hardware parameters of the simulated star sensor are firstly set, as follows: the angle of view is 23° (H) × 23° (V), the focal length is 18 mm, the image size is 1024 × 1024 pixels, the size of each pixel is 10 μm × 10 μm, the sampling frequency is 5 Hz, and the time of exposure is 100 ms. The star images are simulated using the Smithsonian Astrophysical Observatory (SAO) star catalogue. Only the stars with a magnitude no greater than 4 Mv are simulated. All of the images are added by Gaussian noise with 0 mean and 0.001 variance.

**Figure 7:**
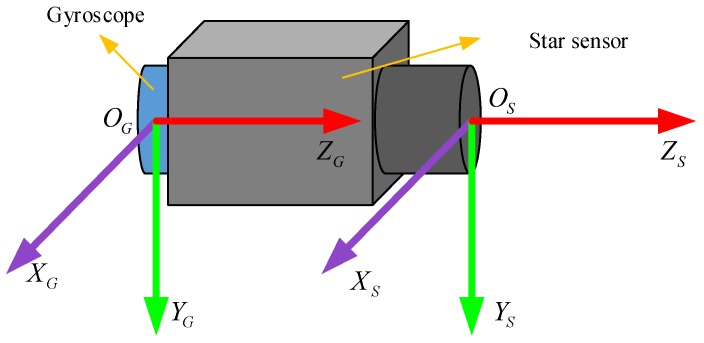
Installation of the star sensor and gyroscope.

It is assumed that the installation parameters between the star sensor and gyroscope are known. As shown in Figure 7, the axis $OZ_{G}$ of the gyroscope coincides with the boresight $OZ_{S}$ of the star sensor, and $OX_{G}$ and $OX_{S}$ are parallel to $OX_{S}$ and $OY_{S}$, respectively.

**Figure 8:**
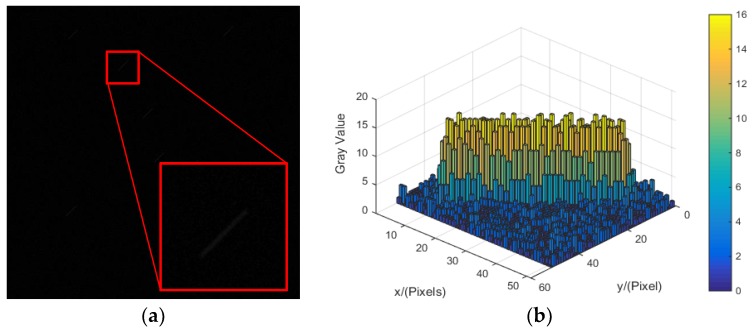
The simulated blur star image and its energy distribution. (**a**) The simulated blur star image. (**b**) The energy distribution of the blur star image.

The actual rotation angular velocity of the star sensor is $\omega_{t}=\left[\omega_{tx},\omega_{ty},\omega_{tz}\right]=\left[4,4,0\right]{}^{\circ}/s$ In this condition, the blurred star image is simulated, as shown in Figure 8.

**Figure 9:**
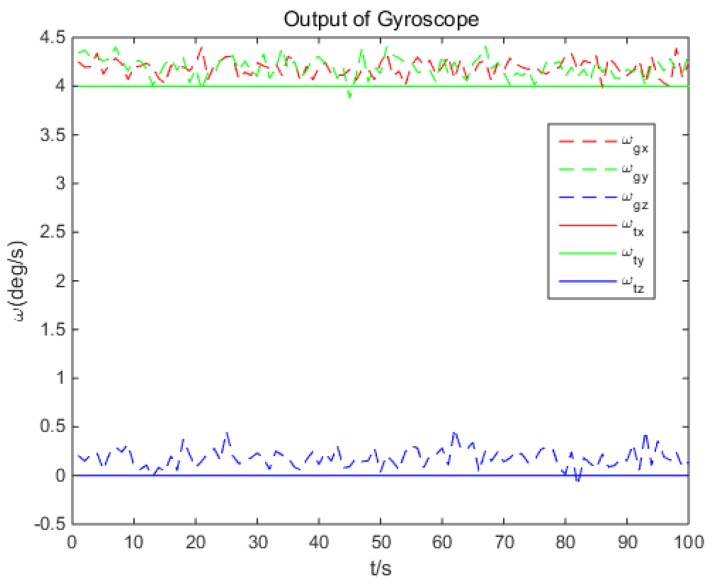
Simulated output data of gyroscope.

The simulated output of gyroscope $\omega_{g}$ is based on $\omega_{t}$, with an additional noise of 0.2°/s bias and 0.1 (°/s)^2^ variance, as shown in Figure 9. The output rate of gyroscope is 100 Hz. 

#### 6.1.2. Results

(1) Comparison of Different Blur Kernel Determination Methods

**Figure 10:**
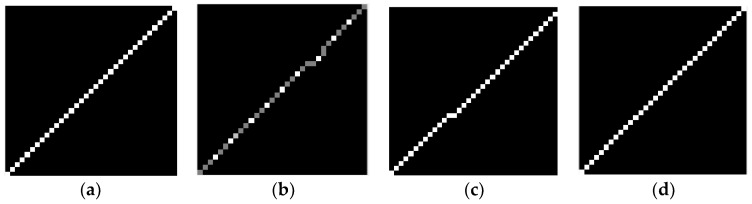
Blur kernel determined by a different method. (**a**) The true blur kernel. (**b**) The blur kernel obtained by gyroscope. (**c**) The blur kernel obtained by improved Radon transform. (**d**) The blur kernel obtained by the proposed method.

This experiment is conducted to verify the error between the blur kernels determined by different methods and the truth. The true blur kernel of the star strip in Figure 8 is shown in Figure 10a. The blur kernel that is calculated directly by the angular velocity from the MEMS gyroscope is shown in Figure 10b. Figure 10c shows the blur kernel by the improved Radon transform from the literature [19]. In the proposed correction method, ***A*** is the identity matrix; the structure information ***T*** is the observer; φ=0.1, ε=0.005, μ=2, $s_{\min}=0.5$; and the corrected blur kernel is shown in Figure 10d. As the shape and size of the blur kernel are both the factors that influence restoration, the mean square error (MSE) and size are chosen as the evaluation indicator to evaluate the similarity to the truth. The MSE of the three blur kernels are $MSE_{gyro}=1.03\times 10^{-5}$,$MSE_{radon}=1.12\times 10^{-5}$, and $MSE_{corrected}=6.11\times 10^{-7}$, and the result of the proposed method is the lowest. As the blur kernels and the truth are not a coincident, the MSEs of the blur kernel obtained by the improved Radon transform method and gyroscope are higher. However, the overall shape is closer to the truth. Additionally, as the width of the blur kernel is equal to one, the length is the size parameter. The length of the three blur kernels are 33 pixels, 35 pixels, and 35 pixels, and the errors are 2 pixels, 0 pixels, and 0 pixels. The proposed method and the improved Radon transform are the same as the truth.

To verify the performance of the different blur kernel determination methods at different angular velocities, these methods are carried out at six groups of angular velocity [3,3,2]°/s, [4,4,0]°/s, [4,4,3]°/s, [5,5,0]°/s, [5,5,4]°/s, and [7,6,5]°/s. 

**Figure 11:**
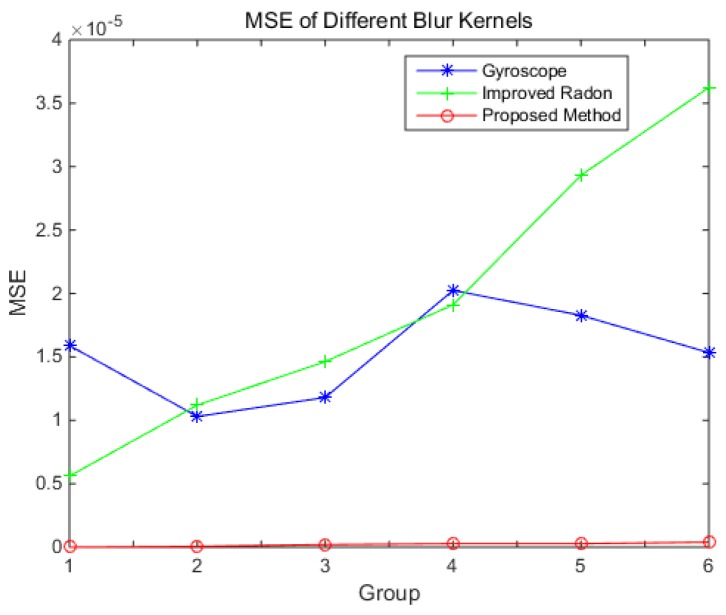
Mean square error (MSE) of the blur kernel by different method.

**Figure 12:**
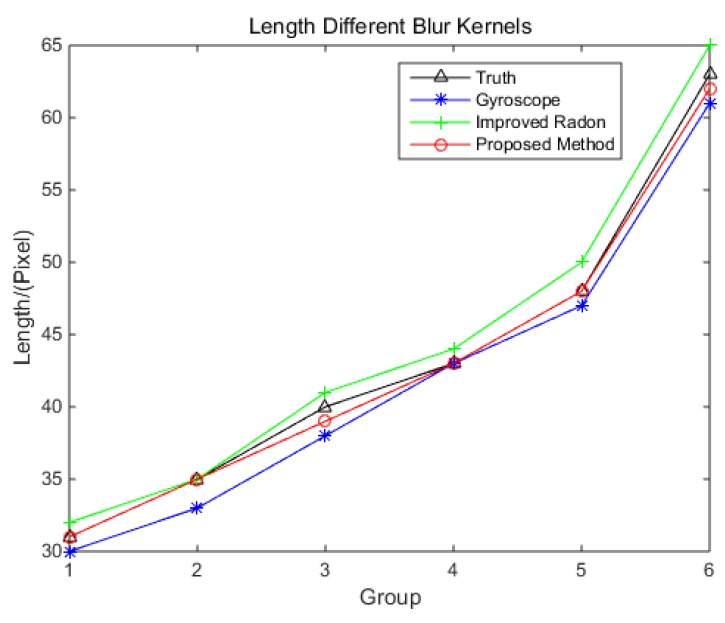
Length of Blur Kernel by different method.

The results of the MSE and length are shown in Figure 11 and Figure 12, respectively.

It can be known that the MSE of the blur kernel from the gyroscope is irregular with the angular velocity. The MSE of the blur kernel from the improved Radon transform method increases with the increase of the angular velocity. The MSE of the blur kernel obtained by the proposed method is the lowest, and it increases slowly with the angular velocity. The length of the blur kernel obtained by the gyroscope method is always shorter than the truth, and it may cause the image to not be fully reconstructed. The length of the blur kernel obtained by the improved Radon transform method is longer than the truth, and the length error increases with the increase of the angular velocity. The length of the blur kernel obtained by the proposed method is similar to the truth. 

From the result above, the error of the blur kernel obtained from the gyroscope is only related to the gyroscope’s bias. The improved Radon transform method is based on the image. When the angular velocity increases, the strip becomes dim, which induces the error of the blur kernel increase. When the star sensor rotates along the boresight (e.g., groups 3 and 5), the error is larger than that without rotating along the boresight. In the proposed method, the blur kernel is corrected by the structure of the star strip. As the energy of the strip has little effect on its structure, the influence of the angular velocity on the error of the blur kernel is slight.

**Figure 13:**
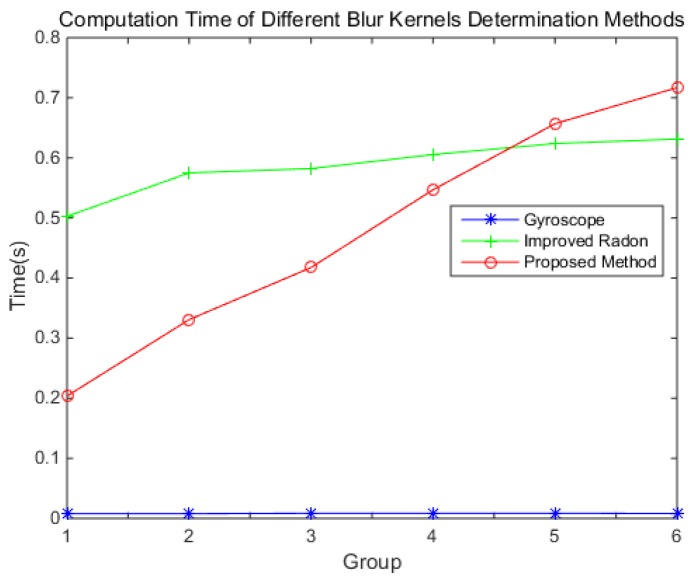
The computation time of different blur kernel determination methods

The computation times of the different blur kernel determination methods are shown in Figure 13.

As the blur kernel determined by the gyroscope is the motion trajectory, it can be calculated very quickly. So, the computation time is short. The improved Radon transform needs image enhancement, so the computation time is longer, and increases with the angular velocity. As the size of the blur kernel increases with the angular velocity, the calculation becomes larger and larger. At a low angular velocity, the proposed method is faster than the improved Radon transform. At a high low-angular velocity, the contrary is the case.

(2) Star Images Reconstruction with Different Blur Kernels

The comparison of the star image reconstructions with different blur kernels are carried out in this experiment. 

**Figure 14:**
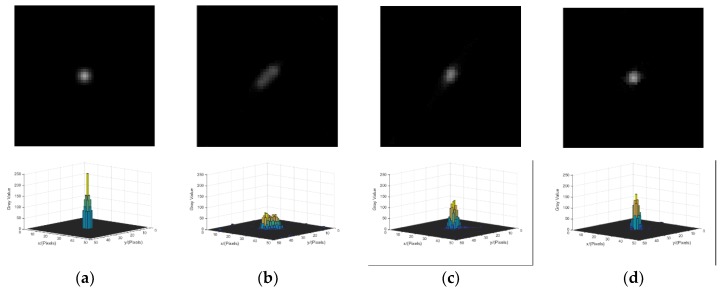
Result of energy distribution after reconstruction with different blur kernels. (**a**) The star spot in the static condition. (**b**) The reconstruction result with blur kernel obtained by the gyroscope. (**c**) The reconstruction result with the blur kernel obtained by the improved Radon transform. (**d**) The reconstruction result with the blur kernel obtained by the proposed method.

The parameters of the reconstruction method are set as $\beta {=10}^{-4}$, θ=0.4, $\alpha_{\min}=10^{-5}$, $\alpha_{m\mathrm{ax}}=10^{5}$, $\alpha_{0}=1.3$, $M_{\alpha }=3$, τ=0.5 and *M *= 1, and the initial value of the iteration is the blur image. As the star image is sparse, it is not necessary to reconstruct the whole image. In order to improve the experiment efficiency, only partial images that contain a stripe are reconstructed. To compare the quality of the result using different reconstruction methods, the energy distribution of the reconstructed star spot, the centroid position error of the star spot, and the peak signal noise ratio (PSNR) of the reconstructed star image is chosen as the evaluation indicator. Figure 14a shows the star spot in the static condition. The reconstructed results with the three blur kernels above are shown in Figure 14b–d, respectively. The result with the blur kernel achieved by the gyroscope is shown in Figure 14b. The reconstructed star is still a stripe with the smaller size, and the energy of the star is not concentrated. The result with the blur kernel achieved by the improved Radon transform is shown Figure 14c. The energy distribution of the reconstructed star spot in Figure 14c is better than that in Figure 14b, but there is still a darker trailing at end of the star spot. The result with blur kernel achieved by the proposed method is shown in Figure 14d. The shape of star spot is approximately a round spot, and the energy distribution of the star is more concentrated which is close to the Gaussian. Therefore, the reconstructed result with the corrected blur kernel is more similar to the star spot in the static condition.

In view of the centroid position error of the star spot, the centroid position of the reconstructed stars is calculated after the star spots segmentation. Table 1 shows the results of the average centroid position error with the three blur kernels at different angular velocities. The first column is the result with the blur kernel obtained by the gyroscope. The second column is the result with the blur kernel obtained by the improved Radon transform. The third column is the result with the blur kernel achieved by the proposed method. The results show that using the corrected blur kernel can obtain a higher accuracy of the centroid position.

**Figure 15:**
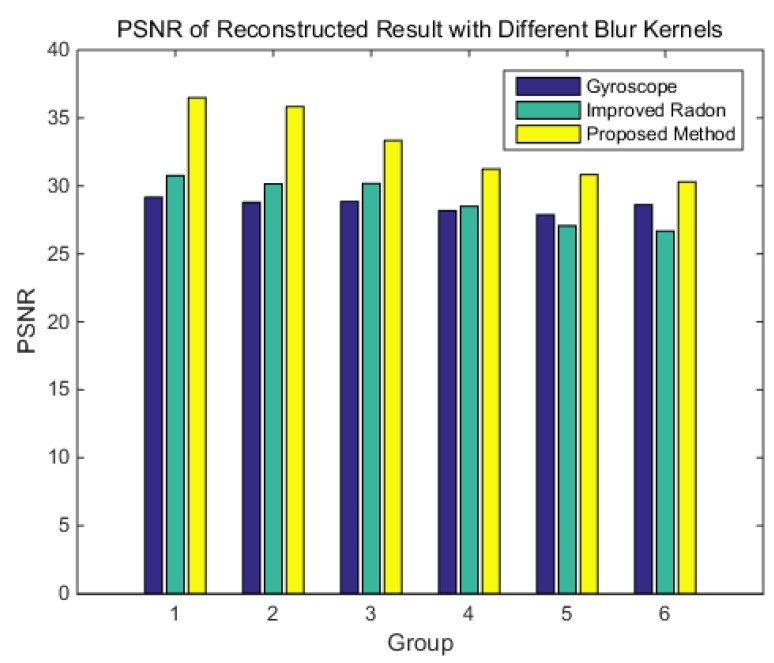
PSNR result after reconstruction by different blur kernels.

PSNR is a parameter used to compare the quality of the reconstructed image with the original image. The greater the PSNR, the more similarity exists between the two images. The result of the PSNR is shown in Figure 15. The results show that the PSNR of the reconstructed image with the blur kernel obtained by the gyroscope is not affected by angular velocity. The PSNR of the reconstructed image with the blur kernel obtained by the improved Radon transform decreases with the increase of the angular velocity. The reconstructed image with the blur kernel obtained by the proposed method are higher than those of the other two methods, that is, the reconstructed star image is closer to the original image. 

The results of the above experiments prove that the proposed blur kernel correction method is effective.

(3) Star Spot Reconstruction with Different Reconstruction Methods

The methods involved in the comparison are the Wiener filter [25], accelerated RL [19] method, and ℓp-regularization [24], and the parameters of the comparison methods are set according to [19,24,25]. All of the reconstruction methods above are carried out with the corrected blur kernel as the uniform condition.

**Figure 16:**
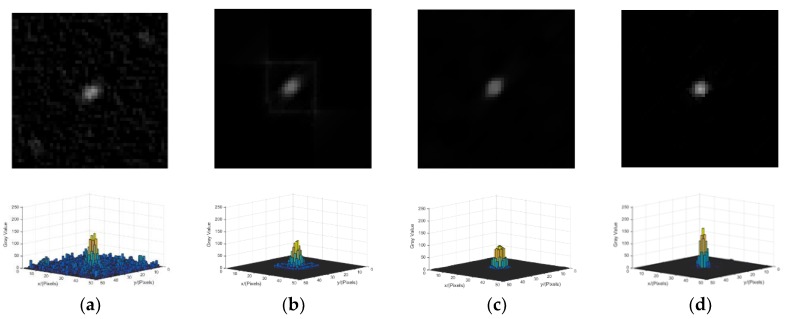
Reconstruction results of different methods. (**a**) Reconstruction result of the Wiener filter. (**b**) Reconstruction result of the accelerated Richardson–Lucy (RL) method. (**c**) Reconstruction result of ℓp-regularization. (**d**) Reconstruction result of the proposed method.

As the Wiener filter can amplify noise, the noise is enhanced in Figure 16a. Figure 16b is the result of accelerated RL method, and the image is reconstructed gradually with the increase in the number of iterations. However, the ringing phenomenon around the spot is more obvious. Figure 16c shows the result of ℓp-regularization, and it is a maximum posteriori probability method that utilizes the prior information of the energy distribution of the star. ℓp-regularization has good robustness to noise, but the overall brightness of the reconstructed star spot is low. The energy concentration of the reconstructed star spot using the ℓp-regularization method is better than that using the accelerated RL method and the Wiener filter. Figure 16d shows the result of the proposed method. The energy concentration and the overall brightness of the reconstructed star spot by using the proposed method are better than the other three methods, and the proposed method is robust to noise.

To verify the performance of the reconstruction methods for the different blurred star images, the comparison experiment is carried out at different angular velocities. The results are evaluated based on the centroid position error, PSNR, and the computational speed. Table 2 is the average centroid position error of the different reconstruction methods at different angular velocities.

The result shows that all of the reconstruction methods can guarantee sub-pixel accuracy at different angular velocities. The first column is the result of the Wiener filter, and the Wiener filter amplifies the noise in the image, which enlarges the error. The second column is the result of the accelerated RL method. The accelerated RL method is robust to noise, but the ringing phenomenon limits its accuracy. The third column is the result of ℓp-regularization, which solves the ringing phenomenon. Its accuracy is higher than the accelerated RL method, however, the overall brightness is low, which limits the accuracy improvement. The forth column is the method proposed in this paper. As the SGP method can always find the optimal solution along the feasible descent direction, using the proposed method can obtain a higher precision centroid position.

**Figure 17:**
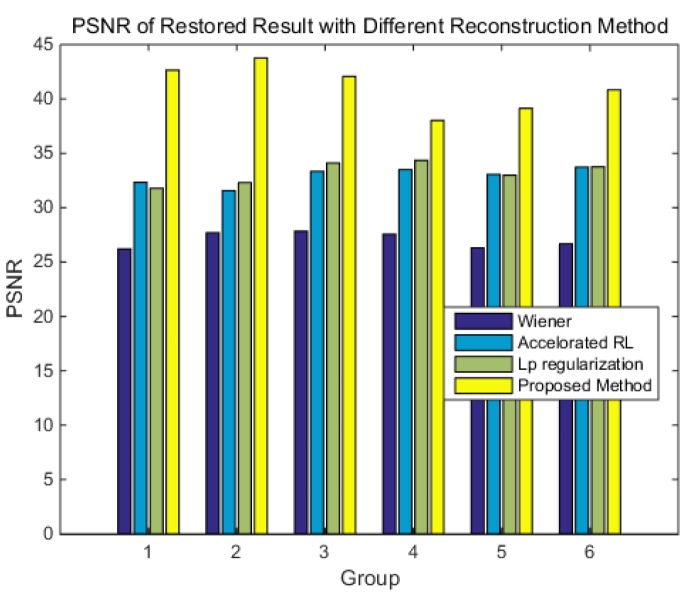
The PSNR of the different reconstruction methods.

Figure 17 is the results of PSNR, the results show that the reconstructed image is more similar to the truth using the method proposed in this paper. Therefore, the results above show that the proposed reconstruction method has better performance.

**Figure 18:**
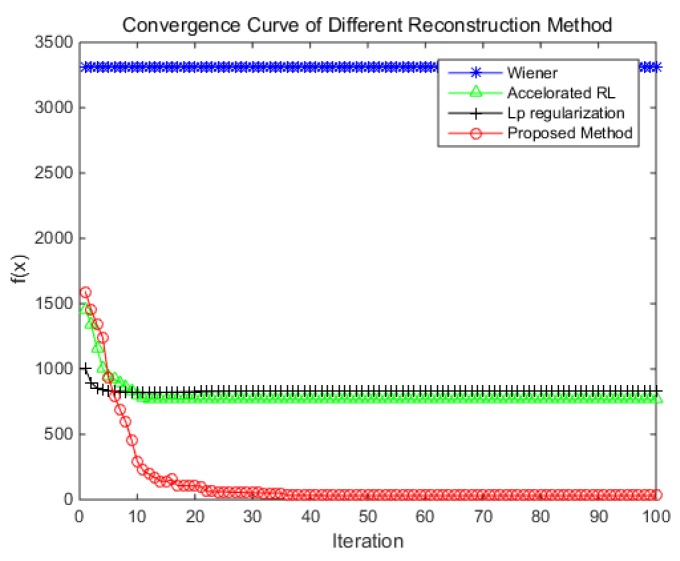
The convergence curve of different methods.

For the computation speed, the convergence rate and the total computation time are chosen as the evaluation indicators. The convergence curve of each method is shown in Figure 18, where the horizontal axis is the number of iterations. The vertical axis is the value of function *f(x)*; the function represents the similarity between the reconstruction result after blur processing and observed blur image. It is defined as follows:

(36) f(x)=‖Ax−b‖

where A is the circulate matrix of the blur kernel; ***x*** is a vector where the entirety of restored image is stacked column by column; ***b*** is the same operation of the observed blur image; ‖·‖ and is the 1-norm of the vector. When the difference between the function values of the current two iterations is less than 1, the objective function is considered to be converged. The experiment result shows that the Wiener filter is a non-iterative method. Therefore, its convergence curve is a horizontal line, and the value of the target function is the maximum. The convergence value of the accelerated RL method and ℓp-regularization are similar. For the accelerated RL method, the objective function value reaches 768.78 after 26 iterations. For ℓp-regularization, the objective function value reaches 831.11 after 28 iterations. For the proposed method, the objective function value reaches 50.78 after 35 iterations. Although it takes slightly more iterations, the value of the objective function is much lower. This shows that the proposed reconstruction result is closer to the truth.

**Figure 19:**
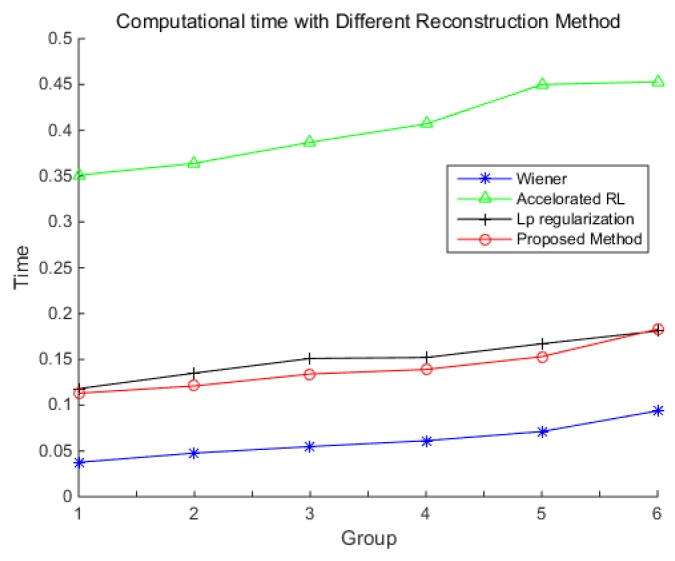
The computation time of different methods.

The computation time of each method at the angular velocities in Table 2 is shown in Figure 19. The result shows that the Wiener filter is the fastest, and the accelerated RL method is the slowest. The proposed method is slightly faster than ℓp-regularization, and offers a speedup of nearly three times more than the accelerated RL method. As the length of the star strip increases with the increase of the angular velocity, the size of the partial image to be reconstructed is larger, which makes the computation time increase, and this can be also found in Figure 18.

The results above show that, in the acceptable calculation time, the result of the proposed reconstruction method is the most similar to the truth, and the position accuracy is the highest. Therefore, this reconstruction method is effective and available for blurred star images.

### 6.2. Star Extraction and Identification in Real Star Image

In this experiment, the proposed algorithm is performed entirely to restore the whole blurred star image. The brightness enhancement of stars, centroid position error, and number of identified stars before and after restoration are the evaluation indicators to verify the proposed algorithm.

**Figure 20:**
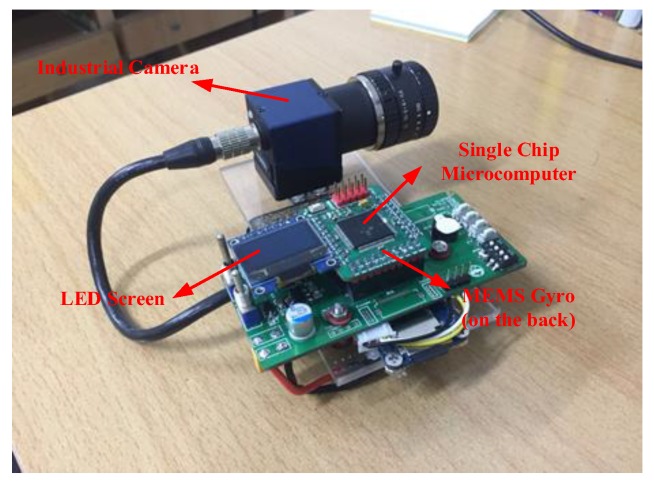
The integrated system.

The star image and angular velocity are obtained by an integrated system that includes an industrial camera (taking the place of the star sensor), a MEMS gyroscope, and a single-chip microcomputer system, as shown in Figure 20. The angular velocity is sampled by the single-chip microcomputer and recorded in a memory card. The star image is sampled by the host computer software on the PC. The synchronization between the camera and the gyroscope is realized by the hardware trigger signal of single-chip microcomputer. The parameters of the integrated system are shown in Table 3.

**Figure 21:**
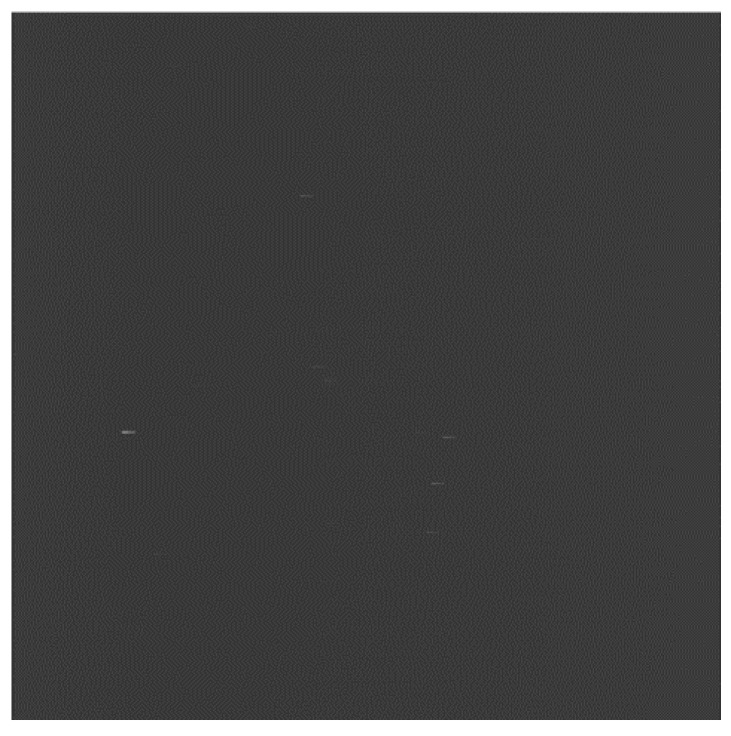
Real blur star image.

The integrated system is fixed on the turntable. When the turntable turns, the camera begins to capture the image, and angular velocity data from the gyroscope is recorded at the same time. The blurred star image is shown in Figure 21. 

**Figure 22:**
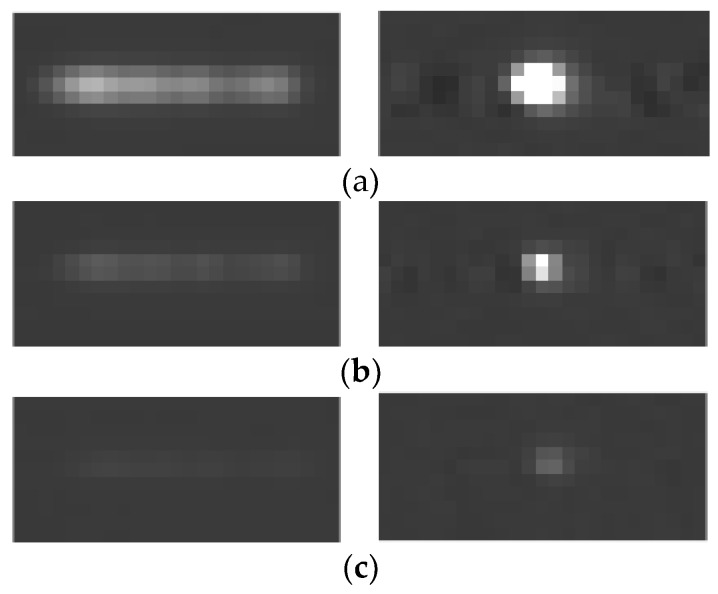
Brightness comparison before and after restoration. (**a**) Brightness comparison of high brightness star strip before and after restoration. (**b**) Brightness comparison of mid brightness star strip before and after restoration. (**c**) Brightness comparison of low brightness star strip before and after restoration.

After restoration, the centroid extraction and star identification are performed with both the blurred star image and the reconstructed star image, and the comparison results are shown in Figure 22, Table 4 and Table 5. 

Figure 22 shows the brightness comparison before and after restoration. In the left column of Figure 22, the star strips are dim before the restoration. Some of the star strips are broken into several pieces and submerged in the background, which cannot be detected. In the right column of Figure 22, the dim pieces of the strip get together after restoration, so the brightness of the star after restoration is enhanced, which becomes easier to detect. The average value of the background pixels is 61. In Figure 22a, the maximum value of the pixel on the stripe is 177, and this value becomes 255 after restoration, and many pixels are saturation. In Figure 22b, the maximum value of pixel on the stripe is 91, and this value become 255 after restoration. In Figure 22c, the maximum value of the pixel on the stripe is 67, and this value become 97 after restoration.

For the centroid position error, it is difficult to determine the truth of the centroid position of the restored star spot. Here, the centroid of the brightest star is chosen as the fiducial point, and the centroid of each star spot relative to the fiducial point is calculated as the relative position. The truth is the relative position in the static star image, and the error with the truth before and after restoration is shown in Table 4.

Table 4 shows that the number of the detectable stars obviously increases after restoration. As the false detection caused by fracture decreases, the centroid accuracy improves.

The detailed information of the identified stars, including the index number and the corresponding magnitude in the Smithsonian Astrophysical Observatory (SAO) Star Catalog of the experiment, is shown in Table 5. It can be observed that the number of identified stars increases from 10 to 24, while the identification rate rises from 37.04% to 88.89%, and the magnitude rises from 4.2 to 5.2. Therefore, the identification rate of the star in the dynamic condition significantly improves after restoration. The performance of the star sensor in the dynamic condition is improved.

## 7. Conclusions

To overcome the problem of the decreased accuracy of the centroid extraction and the failure of the star identification introduced by motion blur, a new image restoration algorithm is investigated in this paper. The new algorithm includes an initial blur kernel calculation, blur kernel correction, and star image reconstruction. In the initial blur kernel calculation, the motion trajectory of the star in the image is calculated, aided by the angular velocity from a MEMS gyroscope. The initial blur kernel is calculated by linear interpolation with the motion trajectory points. In the blur kernel correction, the structure information of the star strip is extracted by Delaunay triangulation. Based on the structure information, the initial blur kernel is corrected by the preconditioned conjugate gradient interior point. In the star image reconstruction, the SGP is used to reduce the computation time. Experiments with simulated star images have been conducted to compare the proposed restoration algorithm with others. The MSE and the length of the blur kernels at different angular velocities show that the proposed blur kernel determination method has a better MSE and length error than those determined by other methods. The same image reconstruction method is carried out for a further comparison of the different blur kernels. The reconstruction results show that the centroid position error, PSNR, energy concentration, and visual quality are better when using the proposed blur kernel determination method. The influence of angular velocity on the blur kernel has also been analyzed. Subsequently, the same blur kernel is used in the comparison of different reconstruction methods. The reconstruction results at different angular velocities show that the proposed reconstruction method is superior in the centroid position error, PSNR, energy concentration, and visual quality. The computation speed of the proposed reconstruction method offers a speedup of nearly three times over the accelerated RL method without introducing unwanted artifacts. In the result of the experiment with a real star image, the accuracy of the centroid has been improved and more stars have been detected after restoration. In addition, the identification rate is up to 88.9%. Therefore, the star sensor can provide attitude information more accurately in the dynamic condition by using the proposed method.

## Figures and Tables

**Table 1:** Average centroid position error after reconstruction with different blur kernels.

Angular Velocity (°/s)			(Pixels)	(Pixels)	(Pixels)
3	3	2	(0.738, 0.880)	(0.189, 0.539)	(0.108, 0.151)
4	4	0	(0.622, 0.854)	(0.490, 0.203)	(0.157, 0.132)
4	4	3	(0.634, 0.759)	(0.138, 0.190)	(0.134, 0.124)
5	5	0	(0.895, 0.617)	(0.692, 0.037)	(0.145, 0.164)
5	5	4	(0.884, 0.819)	(0.389, 0.075)	(0.166, 0.152)
7	6	5	(1.091, 0.906)	(0.535, 0.413)	(0.294, 0.168)
Average error			(0.811, 0.806)	(0.406, 0.243)	(0.167, 0.149)

**Table 2:** The average centroid position error of different reconstruction methods.

Angular Velocity (°/s)			*avg_err_wnr* (pixels)	*avg_err_RL* (pixels)	*avg_err_lp* (pixels)	*avg_err_prop* (pixels)
ω*_x_*	ω*_y_*	ω*_z_*				
3	3	2	(0.567, 0.289)	(0.395, 0.534)	(0.149, 0.211)	(0.108, 0.151)
4	4	0	(0.456, 0.163)	(0.582, 0.592)	(0.102, 0.180)	(0.157, 0.132)
4	4	3	(0.420, 0.124)	(0.219, 0.059)	(0.110, 0.368)	(0.134, 0.124)
5	5	0	(0.379, 0.309)	(0.132, 0.033)	(0.313, 0.419)	(0.145, 0.164)
5	5	4	(0.505, 0.215)	(0.467, 0.259)	(0.452, 0.232)	(0.166, 0.152)
7	6	5	(0.362, 0.816)	(0.495, 0.278)	(0.195, 0.203)	(0.294, 0.168)
Average error			(0.448, 0.319)	(0.382, 0.293)	(0.220, 0.269)	(0.167, 0.149)

**Table 3:** The parameters of integrated system.

Camera Parameters		Gyroscope Parameters	
Focal Length	16 mm	Range	±250°
Image Size	1024 pixels × 1024 pixels	Bias Drift	0.15°/s
Angle of View	22.7° (H) 17.1° (H)	Output Rate	100 Hz
Pixel Size	4.8 μm × 4.8 μm	Total Root Mean Square Noise	0.05°/s
Exposure Time	100 ms		

**Table 4:** Centroid position error before and after restoration.

No	Relative Position in Static Condition				Relative Position in Dynamic Condition			
	Truth				Before Restoration		After Restoration	
	*x* (pixels)	Δ*x* (pixels)	*y* (pixels)	Δ*y* (pixels)	Δ*x_err_* (pixels)	Δ*y_err_* (pixels)	Δ*x_err_* (pixels)	Δ*y_err_* (pixels)
1	171.688	0	600.079	0	0	0	0	0
2	215.723	44.035	773.642	173.563	–3.260	0.096	−0.236	0.081
3	237.442	65.754	78.207	−521.872	−6.461	−0.326	−0.474	−0.236
4	425.399	253.711	263.513	−336.566	−0.660	−0.147	−0.664	−0.249
5	436.188	264.500	507.378	−92.701	−5.028	0.013	−0.527	−0.153
6	459.601	287.913	527.165	−72.914	−3.812	0.184	−0.575	0.005
7	462.934	291.246	730.523	130.444	−4.397	0.511	−0.867	0.248
8	587.362	415.674	600.653	0.574	−6.140	−0.210	−0.315	−0.341
9	604.388	432.700	743.470	143.391	−0.317	0.181	−0.099	−0.094
10	611.949	440.261	673.777	73.698	−0.557	0.304	−0.232	0.088
11	628.240	456.552	607.475	7.396	−0.854	0.085	−0.185	−0.025
12	790.469	618.781	151.494	−448.585	−5.838	−0.456	0.031	−0.054
13	868.238	696.550	646.751	46.472	−5.597	1.380	−0.274	−0.138
14	24.117	−147.571	481.125	−118.954	Failed	Failed	0.745	0.112
15	46.328	−125.360	370.880	−229.199	Failed	Failed	0.094	−0.077
16	102.159	−69.529	625.169	25.090	Failed	Failed	0.427	0.074
17	246.948	75.260	624.517	24.438	Failed	Failed	−0.781	−0.081
18	272.470	100.782	534.993	−65.086	Failed	Failed	−0.542	−0.056
19	344.668	172.980	21.858	−578.221	Failed	Failed	−0.669	−0.070
20	362.997	191.309	485.991	−114.088	Failed	Failed	−0.522	−0.385
21	555.996	384.308	733.717	133.638	Failed	Failed	−0.372	0.247
22	766.269	594.581	399.006	−201.073	Failed	Failed	−0.492	0.205
23	824.985	653.297	846.490	246.411	Failed	Failed	−0.220	0.209
24	956.433	784.745	227.671	−372.408	Failed	Failed	0.608	0.709

**Table 5:** Star identification before and after restoration.

No	Star Identification in Static Condition				Star Identification in Dynamic Condition			
					Before Restoration		After Restoration	
	*x* (pixels)	*y* (pixels)	Index	Mag	Index	Mag	Index	Mag
1	171.688	600.079	131907	0.3	131907	0.3	131907	0.3
2	868.238	646.751	113271	0.6	113271	0.6	113271	0.6
3	611.949	673.777	132346	1.8	Failed	Failed	132346	1.8
4	628.240	607.475	132444	2	132444	2	132444	2
5	425.399	263.513	132542	2.2	132542	2.2	132542	2.2
6	604.388	743.470	132220	2.5	132220	2.5	132220	2.5
7	215.723	773.642	131794	2.9	131794	2.9	131794	2.9
8	462.934	730.523	132071	3.4	132071	3.4	132071	3.4
9	237.442	78.207	150801	3.7	Failed	Failed	150801	3.7
10	246.948	624.517	131952	3.7	132406	3.8	131952	3.7
11	824.985	846.490	112921	3.7	112921	3.7	112921	3.7
12	344.668	21.858	150957	3.8	Failed	Failed	150957	3.8
13	587.362	600.653	132406	3.8	Failed	Failed	132406	3.8
14	790.469	151.494	133012	4.1	Failed	Failed	133012	4.1
15	272.470	534.993	132067	4.2	Failed	Failed	132067	4.2
16	459.601	527.165	132320	4.2	132320	4.2	132320	4.2
17	46.328	370.880	150340	4.3	Failed	Failed	150340	4.3
18	102.159	625.169	131824	4.3	Failed	Failed	131824	4.3
19	24.117	481.125	150223	4.5	Failed	Failed	150223	4.5
20	362.997	485.991	132222	4.6	Failed	Failed	132222	4.6
21	436.188	507.378	132301	4.7	Failed	Failed	132301	4.7
22	766.269	399.006	132732	4.7	Failed	Failed	132732	4.7
23	555.996	733.717	132176	5	Failed	Failed	132176	5
24	956.433	227.671	133118	5.2	Failed	Failed	133118	5.2
25	443.191	507.327	132321	5.2	Failed	Failed	Failed	Failed
26	506.795	828.620	132024	5.6	Failed	Failed	Failed	Failed
27	16.063	489.609	150206	5.9	Failed	Failed	Failed	Failed

## Appendix A

An equality constrain z=Ax-y is introduced into Equation (14). The Equation (14) is transformed into the following:

(A1) $$\begin{array}{l} \mathrm{minimize}\;z^{T}z+\varphi {\left\|x\right\|}_{1}\\ \mathrm{subject}\;\mathrm{to}\;z=Ax-y \end{array}$$

For the dual feasible point $\upsilon \in {\mathbb{R}}^{m}$, the Lagrangian is as follows:

(A2) $$L\left(x,y,\upsilon \right)=z^{T}z+\lambda {\left\|x\right\|}_{1}+\upsilon^{T}\left(Ax-y-z\right)$$

The dual function of (A1) as the minimum value of Lagrangian over ***x*** and ***z*** is as follows:

(A3) $$\underset{x,z}{\inf}L\left(x,z,\upsilon \right)=\underset{x,z}{\inf}\left(z^{T}z+\lambda {\left\|x\right\|}_{1}+\upsilon^{T}\left(Ax-y-z\right)\right)$$

As the L(x,z,υ) is a convex quadratic function of ***z***, the minimizing ***z*** from the optimality condition as follows:

(A4) $$\nabla_{z}L\left(x,z,\upsilon \right)=2z+\upsilon$$

which yields $z=\frac{1}{2}\upsilon$. According to the literature [38] (Equation (5.2) in page 216)

(A5) $$-\frac{1}{4}\upsilon^{T}\upsilon +\lambda {\left\|x\right\|}_{1}+\upsilon^{T}\left(Ax-y\right)\leq \inf\left\{z^{T}z++\varphi {\left\|x\right\|}_{1}|z=Ax-y\right\}$$

According to the literature [38] (Equation (5.12) in pages 221–222), the dual function for problem (A1) is as follows:

(A6) $$\underset{x,z}{\inf}L\left(x,z,\upsilon \right)=\{\begin{array}{cc} \begin{array}{l} -\frac{1}{4}\upsilon^{T}\upsilon -\upsilon^{T}y,\\ -\infty , \end{array} & \begin{array}{l} \left|\left(\left(A^{T}\upsilon \right)\right)\right|\leq \lambda_{i}\\ \mathrm{else} \end{array} \end{array}$$

where $G\left(\upsilon \right)=-\frac{1}{4}\upsilon^{T}\upsilon -\upsilon^{T}y$ is the dual objective of (A1). According to the literature [39], the dual feasible point is multiplied by a step size *s*.

(A7) $$\begin{array}{l} \upsilon =2s\left(Ax-y\right)\\ s=\min\left\{\varphi /\left|2{\left(A^{T}Ax\right)}_{i}-2y_{i}\right|,i=1,2,...,m\right\} \end{array}$$
